# Anti-Cancer Effects of Queen Bee Acid (10-Hydroxy-2-Decenoic Acid) and Its Cellular Mechanisms against Human Hepatoma Cells

**DOI:** 10.3390/molecules28041972

**Published:** 2023-02-19

**Authors:** Zafer Saad Al Shehri, Abdullah D. Alanazi, Sultan F. Alnomasy

**Affiliations:** 1 Department of Medical Laboratories, College of Applied Medical Sciences, Shaqra University, KSA, Al Dawadmi 1678, Saudi Arabia; 2 Department of Biological Sciences, Faculty of Science and Humanities, Shaqra University, Ad-Dawadimi 11911, Saudi Arabia; 3 Department of Medical Laboratories Sciences, College of Applied Medical Sciences, Shaqra University, Al-Quwayiyah 19257, Saudi Arabia

**Keywords:** royal jelly, natural products, apoptosis, cell viability

## Abstract

Background: Hepatocellular carcinoma (HCC) is the most common form of liver cancer that occurs in hepatocytes. Although many chemical drugs, e.g., cisplatin, methotrexate, taxis, and doxorubicin are used to treat HCC, there have been numerous reports related to the side effects of these drugs (e.g., emerging drug resistance, bone marrow failure, and gastrointestinal disorders). These issues led scientists to search for the novel anti-cancer drugs, mainly in natural products with greater efficiency and less toxicity. The current survey was intended to assess the anti-cancer effects of queen bee acid (10-Hydroxy-2-Decenoic Acid, 10-HDA) and its cellular mechanisms against the human hepatoma cell line HepG2. Materials and Methods: The MTT (3-[4,5-dimethylthiazol-2-yl]-2,5 diphenyl tetrazolium bromide) assay was used to evaluate the effect of 10-HDA on the viability of HepG2 cells. The initial and late apoptosis in the HepG2 cells treated with 10-HDA were assessed by the Annexin-V (AV) assay. The level of the gene and protein expression of some apoptosis genes (e.g., caspase-3, Bcl-2-associated X protein (BAX), and B-cell lymphoma protein 2 (Bcl-2)), Poly (ADP-ribose) polymerases (PARP), and miRNA-34a (miR-34a), were measured by real-time PCR and Western blot. Results: The obtained findings revealed that HepG2 cell viability was markedly reduced (*p* < 0.01) following exposure to 10-HDA in a dose-dependent matter. The calculated half maximal cytotoxic concentration (CC50) value of 10-HDA was 59.6 µg/mL for HepG2 cells, while this value for normal THLE-3 cells was 106.4 µg/mL. We found that 10-HDA markedly elevated (*p* < 0.01) the percentage of necrotic and apoptotic cells from 0.94 to 9.7 and 27.6%, respectively. The real-time PCR results showed that the expression levels of the caspase-3, Bax, and miR-34a genes were significantly (*p* < 0.001) elevated. Contrary to these results, a significant (*p* < 0.01) reduction in the expression level of the Bcl2 gene was observed. The levels of protein expression of Caspase-3, PARP, and Bax were markedly elevated following exposure of HepG2 cells to 10-HDA at ¼ CC50, ½ CC50, and CC50. The level of protein expression of Bcl-2 was markedly reduced following exposure of HepG2 cells to 10-HDA at ¼ CC50, ½ CC50, and CC50 (*p* < 0.01). Conclusion: The current results confirmed the potent in vitro cytotoxic effects of 10-HDA on HepG2 cells with no significant cytotoxic effects on normal cells. Although its mechanisms of action have not been fully studied, the induction of apoptosis via different pathways was determined as one of the principle mechanisms of action of 10-HDA against HepG2 cells. Nevertheless, additional surveys must be performed to clearly understand the mechanisms of action and safety of this fatty acid.

## 1. Introduction 

Hepatocellular carcinoma (HCC) is the most common form of liver cancer that occurs in hepatocytes and most often occurs after a person is infected with hepatitis B, or C or develops cirrhosis [1]. Hepatocellular carcinoma is the 3rd most common cause of cancer deaths around the world, with a relative 5-year survival rate of about 18% [2]. HCC, like most other cancers, occurs when a mutation disrupts the cell division cycle leading to excessive proliferation of cells [1,2]. It has been proven that the production of active oxygen radicals such as superoxide anion radical, hydroxyl radical, and hydrogen peroxide (H_2_O_2_) are important factors in various pathological conditions, such as cancer [3]. HCC is reported as the 5th most prevalent cancer, with a million cases of the disease being reported around the world each year [2]. Several factors have been reported as risk factors for this disease, e.g., mutagenic drugs, high alcohol consumption, and infections [3]. Today, there are various methods such as chemotherapy with synthetic drugs, radiotherapy, immunotherapy, and surgery to improve the prognosis and long-term survival of HCC patients [4]. Although many chemical drugs, e.g., cisplatin, methotrexate, taxis, and doxorubicin, are used to treat HCC, there have been numerous reports related to the side effects of these drugs (e.g., emerging drug resistance, bone marrow failure, and gastrointestinal disorders) [5]. 

Doxorubicin (DOX) or adriamycin, an anthracycline, is one of the preferred synthetic chemotherapy agents which is broadly used in the treatment of various types of cancers, e.g., liver, breast, ovary, prostate, and stomach cancers [5]. This drug is able to reduce or inhibit the growth and development of cancer cells through blocking an enzyme called topoisomerase, a key enzyme in the division and grow of cells. Recent reports showed that this drug is commonly associated with serious side effects such as increased risk of infections due to leukopenia, bruising, bleeding gums, and nose bleeds because due to thrombocytopenia, diarrhea, constipation, fever, breathlessness, and appetite loss [5,6]. These issues have led scientists to search for novel anti-cancer drugs, mainly in natural products, that have greater efficiency and less toxicity [7].

In recent times, growing attention has been given to the benefits of food and diets for treating and preventing various diseases and conditions such as cancers [8]. Bee products (honey, royal jelly (RJ), and propolis) are famous valuable foods that have many health-promoting and therapeutic benefits [9]. Analyses of the bioactive compounds in RJ showed the presence of protein, lipids, flavonoids, and polyphenols with a number of therapeutics activities, e.g., anti-inflammatory, antioxidant, anti-cancer, and antimicrobial properties [9,10]. Among these bioactive compounds, the lipids comprise nearly 5–20% of RJ volume, with >90% of these lipids reported as hydroxyl or dicarboxylic fatty acids [11]. Queen bee acid (10-Hydroxy-2-Decenoic Acid, 10-HDA) is well-known as a lipid constituent or fatty acid in RJ [12,13] that exhibits numerous pharmacological and therapeutic effects, e.g., antioxidant, anti-inflammatory, anti-tumor, immune boosting, and antimicrobial activities [14,15,16]. Recently, Alnomasy et al. [17] showed the promising anti-cancer effects of royal jelly against human hepatoma cells (HepG2) via the induction of apoptosis through different pathways; however, the anti-cancer effects of specific compounds from RJ (such as 10-HDA) against human hepatoma cells have not yet been determined. The current survey intended to assess the anti-cancer effects of 10-HDA and its cellular mechanisms against the human hepatoma cell line HepG2 in comparison with doxorubicin.

## 2. Results

### 2.1. Cell Viability

**Figure 1:**
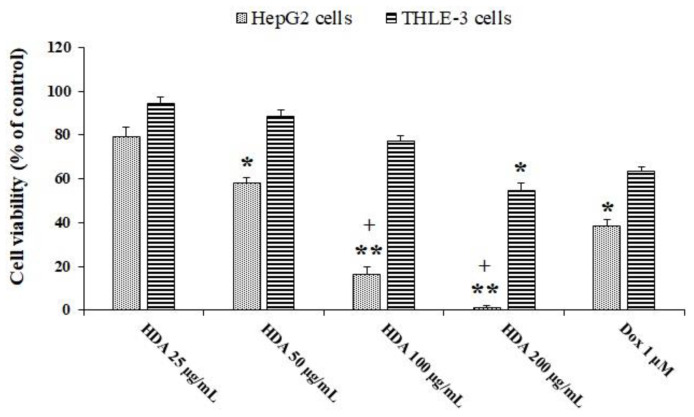
Viability of HepG2 and THLE-3 cells exposed to different concentrations of queen bee acid (10-Hydroxy-2-Decenoic Acid, 10-HDA) or 1 μM doxorubicin (DOX) for two days. * *p* < 0.05, ** *p* < 0.001 compared with the control; + *p* < 0.001 compared with DOX. The findings revealed that HepG2 cell viability was markedly reduced (*p* < 0.01) following exposure to 10-HDA in a dose-dependent manner.

The MTT (3-[4,5-dimethylthiazol-2-yl]-2,5 diphenyl tetrazolium bromide) assay was used to evaluate the cytotoxic effect of 10-HDA on the cell viability of HepG2 and THLE-3 cells. The findings revealed that HepG2 cell viability was markedly reduced (*p* < 0.01) following exposure to 10-HDA in a dose-dependent manner (Figure 1). The calculated CC50 value of 10-HDA was 59.6 µg/mL for HepG2 cells and 106.4 µg/mL for normal THLE-3 cells. The findings also showed that 1 μM DOX reduced the viability of THLE-3 and HepG2 cells by 63.4% and 38.2%, respectively. 

### 2.2. Morphological Study

**Figure 2:**
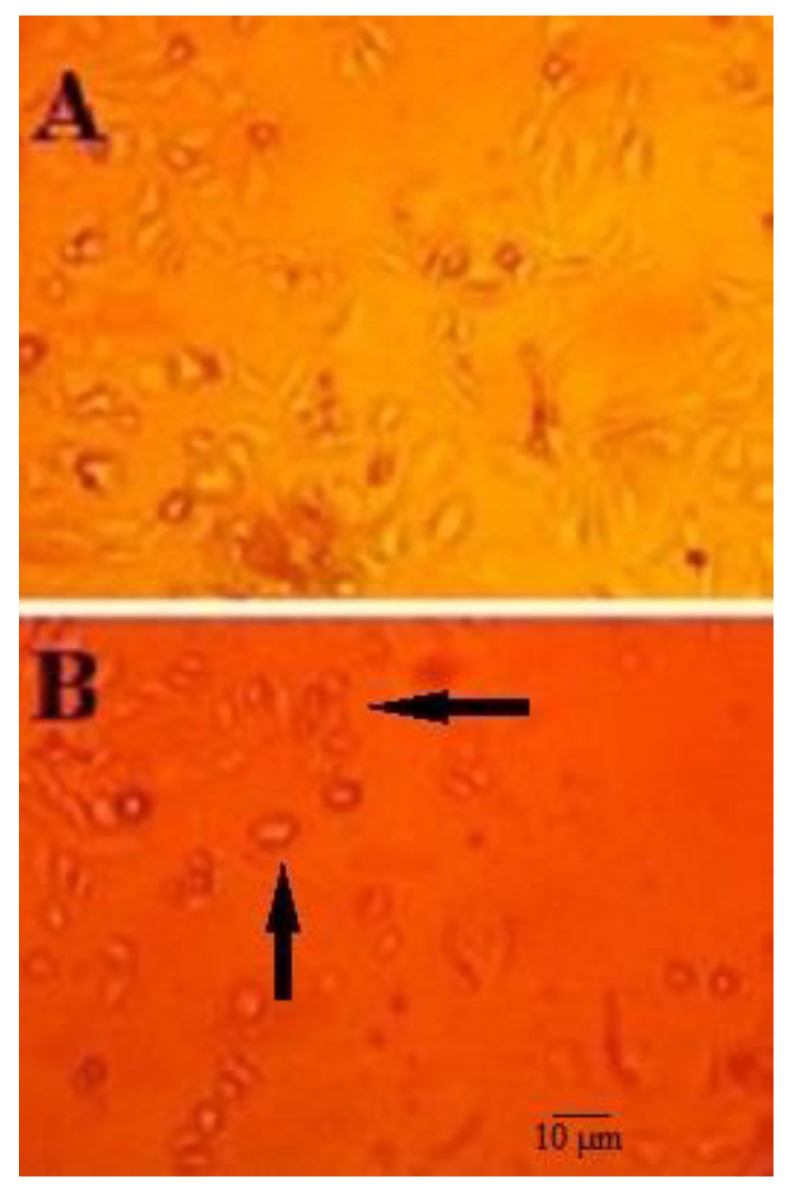
Morphological changes in the structure of HepG2 cells treated with queen bee acid (10-Hydroxy-2-Decenoic Acid, 10-HDA) at ½ the half maximal cytotoxic concentration (CC50) concentration for two days (**A**) and HepG2 cells that were not treated with 10-HDA (**B**). After exposure of HepG2 cells to 10-HDA (½ CC50 for two days), the cells showed smaller sizes and round shapes with marked cytoplasmic shrinkage (black arrows), while HepG2 cells with no treatment displayed a spindle shape with the same size.

As shown in Figure 2, following exposure of HepG2 cells to 10-HDA (at ½ CC50 for two days), the cells showed smaller sizes and round shapes with marked cytoplasmic shrinkage, while HepG2 cells with no treatment displayed a spindle shape with the same size.

### 2.3. Annexin-V Assay

**Figure 3:**
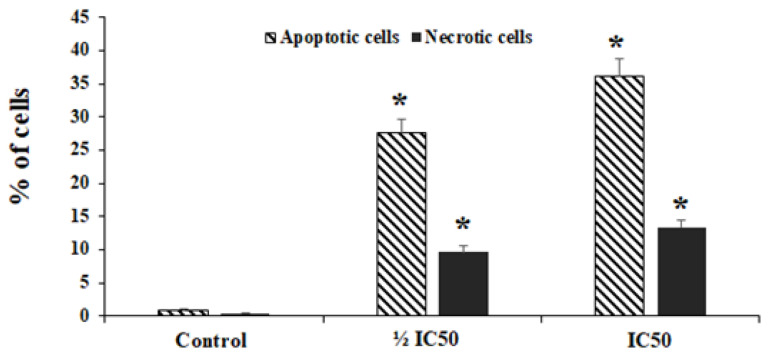
The rate of apoptosis and necrotic cells following treatment with the queen bee acid (10-Hydroxy-2-Decenoic Acid, 10-HDA) at ½ the half maximal cytotoxic concentration (CC50) and CC50. Data shown as mean ± SD (n = 3). * *p* < 0.001. We found that 10-HDA markedly elevated (*p* < 0.01) the number of necrotic and apoptotic cells. Meanwhile, exposure to 10-HDA at CC50 markedly elevated (*p* < 0.001) the number of necrotic and apoptotic cells.

Annexin-V assay was used to assess the level of early and late apoptosis in HepG2 cells treated with 10-HDA. We found that 10-HDA (½ CC50 for two days) markedly elevated (*p* < 0.01) the number of necrotic and apoptotic cells from 0.94% to 9.7 and 27.6%%, respectively. Meanwhile, exposure to 10-HDA at CC50 markedly elevated (*p* < 0.001) the number of necrotic and apoptotic cells from 0.94% to 13.4% and 36.2%, respectively (Figure 3).

### 2.4. Evaluating Apoptosis-Related Gene Expression

**Figure 4:**
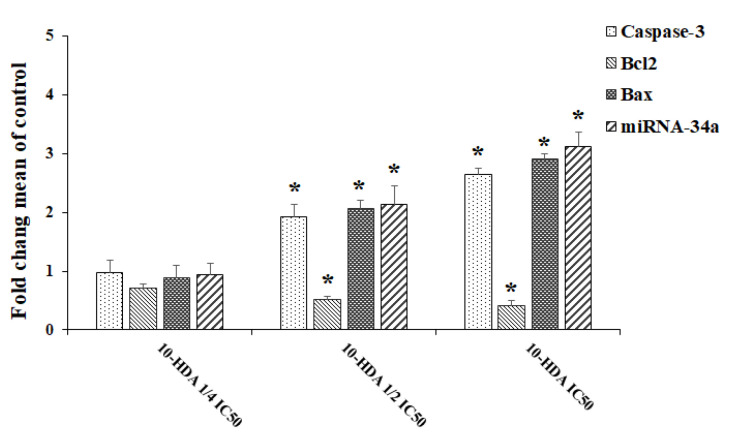
The levels of gene expression of some apoptosis genes (Caspase-3, Bax, and Bcl-2) and the miR-34a gene in HepG2 cells after exposure to queen bee acid (10-Hydroxy-2-Decenoic Acid, 10-HDA) at ¼ the half maximal cytotoxic concentration (CC50), ½ CC50, and CC50. Data shown as mean ± SD (n = 3). * *p* < 0.001. Real-time PCR results showed that the expression levels of the caspase-3 and Bax genes were significantly upregulated following exposure to 10-HDA. Contrary to these results, the exposure of HepG2 cells to 10-HDA caused a significant (*p* < 0.01) reduction in the expression levels of the Bcl2 and miR-34a genes.

Real-time PCR was used to determine the levels of gene expression of miR-NA-34a and some apoptosis genes, e.g., caspase-3, Bax, and Bcl2. The findings showed that the expression level of caspase-3 gene was markedly (*p* < 0.001) elevated by 1.93- and 2.64-fold following exposure to 10-HDA at ½ CC50 and CC50, respectively. The mRNA levels for the Bax gene were increased 2.06- and 2.91-fold following exposure to 10-HDA at ½ CC50 and CC50, respectively. Contrary to these results, the exposure of HepG2 cells to 10-HDA at ½ CC50 and CC50 resulted in a significant (*p* < 0.01) reduction in the expression level of the Bcl2 gene (Figure 4). The results also showed that the exposure of HepG2 cells to 10-HDA at ½ CC50 and CC50 caused a significant (*p* < 0.001) elevation in the expression levels of the miR-34a gene (Figure 4).

### 2.5. Western Blot

**Figure 5:**
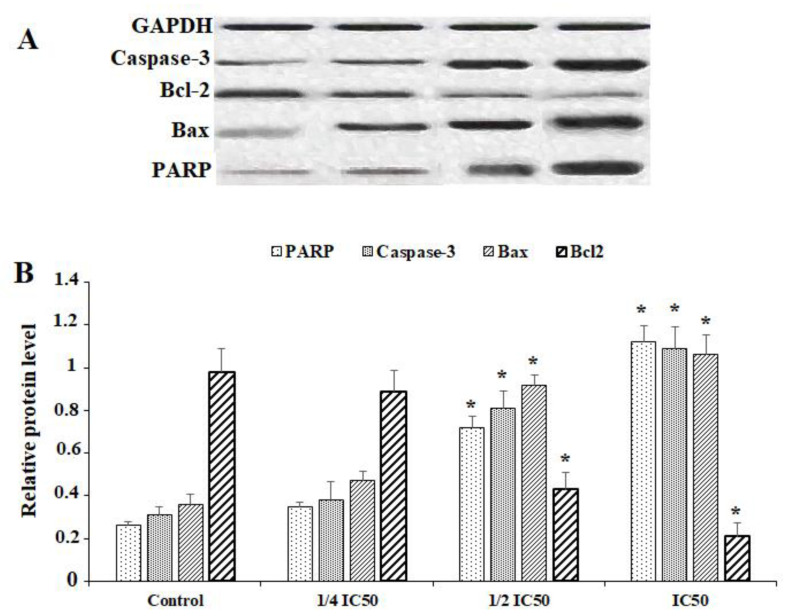
Western blot (**A**) and relative protein expression levels (**B**) of some apoptosis genes (Caspase-3, Bcl-2-associated X protein (BAX), and B-cell lymphoma protein 2 (Bcl-2)) and poly [ADP-ribose] polymerase (PARP) in HepG2 cells after exposure to queen bee acid (10-Hydroxy-2-Decenoic Acid, 10-HDA) at ¼ the half maximal cytotoxic concentration (CC50), ½ CC50, and CC50. Data shown as mean ± SD (n = 3). * *p* < 0.001. Based on the results of the Western blot assay, the levels of protein expression of Caspase-3, PARP, and Bax was markedly elevated following exposure of HepG2 cells to 10-HDA. In contrast to these results, the level of protein expression of Bcl-2 was markedly reduced following exposure of HepG2 cells to 10-HDA.

For evaluation of the effect of 10-HDA on the protein expression levels of PARP, Caspase-3, Caspase-9, Bcl2, Bax, and GADPH, the cells were treated with various concentrations of 10-HDA for 24 h. Following lysing of the cells, the protein was extracted and used for Western blots. Based on the results of the Western blot assay, the levels of protein expression of Caspase-3, PARP, and Bax were markedly elevated following exposure of HepG2 cells to 10-HDA at ¼ CC50, ½ CC50, and CC50. In contrast to these results, the level of protein expression of Bcl-2 was markedly reduced following exposure of HepG2 cells to 10-HDA at ¼ CC50, ½ CC50, and CC50 (*p* < 0.01) (Figure 5).

## 3. Discussion

Liver cancer is a global health concern and its frequency is rising around the world. It is expected that, by 2025, >1 million people will be affected by liver cancer annually [1,2]. At present, systemic treatments, such as immune checkpoint inhibitors (ICIs), tyrosine kinase inhibitors (TKIs), and monoclonal antibodies, are the main therapies for HCC [3]. Although many chemical drugs, e.g., cisplatin, methotrexate, taxis, and doxorubicin, are used to treat HCC, there have been numerous reports related to the side effects of these drugs (e.g., emerging drug resistance, bone marrow failure, and gastrointestinal disorders) [5,6]. These issues have led scientists to search for novel anti-cancer drugs, mainly in natural products, that have greater efficiency and less toxicity [7]. Based on previous investigations of the chemical composition of RJ, free fatty acids, such as the hydroxyl and dicarboxylic acids, are the key lipids of dry RJ [9,10]. 10-HDA is one of the principle fatty acids in RJ and displays a number of pharmacological and therapeutic effects, e.g., antioxidant, anti-inflammatory, anti-tumor, boosting immunity system, and antimicrobial activities [14,15,16,17]. Here, we assessed the anti-cancer effects of 10-HDA and investigated its cellular mechanisms against the human hepatoma cell line HepG2 in comparison with DOX. Our results indicated that HepG2 cell viability was markedly reduced (*p* < 0.01) following exposure to 10-HDA in a dose-dependent manner. The calculated CC50 value of 10-HDA was 59.6 µg/mL for HepG2 cells and 106.4 µg/mL for normal THLE-3 cells. 

It has been proven that heterocyclic analogs with a spiro-thiazolidines structure display potent pharmacological effects against various types of cancers [18]. Recently, Alnomasy et al. (2022) showed the promising anti-cancer effects of royal jelly against HepG2 with a CC50 value of 1.13 mg/mL; in addition, RJ upregulated expression of the miR-34a, Caspase-3, and Bax genes and proteins [17]. Albalawi et al. (2021) reported that 10-HDA at concentrations of 2.5 and 5 mg/kg (along with cyclophosphamide) displayed potent antitumor activity against Ehrlich solid tumors, a murine mammary adenocarcinoma, in mice [19]. Lin et al. (2020) showed the promising cytotoxic effects of 10-HDA against some human lung cancer cell lines (e.g., A549, NCI-H23, and NCI-H460) with CC50 values ranging from 22.6 to 44.7 µg/mL, while having no significant cytotoxicity on normal human lung fibroblasts [20]. Yang et al. (2018) demonstrated that the production of pro-inflammatory cytokines, such as interleukin (IL)-8, IL-1β, and tumor necrosis factor-alpha (TNF-α), in human colon cancer cells (WiDr cells) was significantly decreased by 10-HDA. IL-8 was dramatically reduced by treatment with 10-HDA at 3 mM, whereas IL-1β and TNF-α were also reduced. 10-HDA elevated IL-1ra levels in a dose-dependent manner. The nuclear factor kappa B (NF-κB) pathway is the main response to prototypical pro-inflammatory cytokines, and NF-κB was reduced after 10-HDA treatment [21]. Townsend et al., revealed that 10-HDA at a concentration of 1.5 mg/mL repressed the formation of tumors by some cancer cells including mouse leukemia, 6CSHED lymphosarcoma, TAS mammary carcinoma, and Ehrlich carcinoma [22,23]. Another study conducted by Filipi et al. (2015) showed that 10-HDA had a potent anti-proliferative effect at a concentration of 37.5 μmol/mL against human colorectal adenocarcinoma cells [24]. Regarding to the anti-cancer mechanisms of natural fatty acids, previous studies reported that these compounds display anti-cancer effects through the alteration of the response of immune system to cancer cells through the suppression of arachidonic acid (AA, 20:4n-6)-derived eicosanoid biosynthesis; the modification of metabolism, cell growth, and differentiation; and change in estrogen metabolism, which leads to reduced free radical generation and apoptosis induction [25].

It has been proven that the development and progress of cancer is regularly complemented with an abnormal increase and resistance to apoptosis in cancer cells [26]. Apoptosis is accompanied by unexpected morphological alterations, chromatin condensation, and deoxyribonucleic acid (DNA) destruction [26]. There are several genes and proteins which are involved in the apoptosis process, such as caspase enzymes and Bcl-2 family members [27]. One of the main Caspases genes in apoptotic process is Caspase 3, which directly is linked to apoptosis mechanisms, e.g., DNA destruction and chromatin condensation [26,27]. Contrary to this issue, Bcl-2 proteins are considered the main factors for inhibiting the apoptosis process by means of hindering cytochrome *c* discharge from mitochondria [28]. One of the key tumor suppressor biomarkers is miR-34a, that has been applied to the study metastasis, drug resistance, and cancer prognosis [29]. It has been proven that miR-34a downregulation can lead to the disruption of some critical processes such as apoptosis and cell growth [30,31]. One of the principle enzymes, PARP, is considered an important enzyme involved in some vital cell processes, e.g., synthesis of chromatin, replication, and DNA synthesis [32]. Based on recent reports, upregulation and increased activity of this enzyme lead to activation of specific apoptosis processes characterized by mitochondrial destruction, disturbance of calcium balance in the cell, and stimulation of the secretion of apoptotic factors [32,33]. Our results exhibited that HepG2 cells treated with various concentrations of RJ (0.25, 0.5, 1, and 2 mg/mL) increased PARP expression, indicating that RJ might induce cell death through a specific apoptosis pathway.

The real-time PCR and Western blot results showed that the expression levels of caspase-3, Bax, PARP, and miR-34a genes and proteins were markedly (*p* < 0.001) elevated following exposure to 10-HDA at ½ CC50 and CC50. Contrary to these results, the exposure of HepG2 cells to 10-HDA resulted in a significant (*p* < 0.01) reduction in the expression levels of the Bcl2 gene and protein. In addition, we found that 10-HDA markedly elevated (*p* < 0.01) the number of necrotic and apoptotic cells using the Annexin-V assay.

Recently, Albalawi et al. (2021) reported that following the treatment of the Ehrlich solid tumor-bearing mice with 10-HDA at doses of 2.5 and 5 mg/kg, especially in combination with cyclophosphamide, the expression levels of the caspase-3 and Bax genes were markedly increased in the tumor tissues in the mice. They also found that the expression level of Bcl2 was considerably reduced in the tumor after treatment of the Ehrlich solid tumor-bearing mice with 10-HDA at doses of 2.5 and 5 mg/kg, especially in combination with cyclophosphamide [19]. Furthermore, in the study carried out by Lin et al. (2020), the findings revealed that 10-HDA induced apoptosis in human lung cancer cell lines (A549 cells) through modulating mitochondrial-associated apoptosis and caused cell cycle arrest at the G0/G1 phase in a time-dependent manner [20].

## 4. Materials and Methods

### 4.1. Chemicals

10-HDA (C_10_H_18_O_3_ with purity higher than 99%), thiazolyl blue tetrazolium bromide powder [3-(4,5-dimethyl-2-thiazolyl)-2,5-diphenyl-2-H-tetrazolium bromide); MTT], fetal bovine serum (FBS), and Dulbecco’s modified Eagle’s medium (DMEM) were purchased from Sigma Aldrich (Louis, MO, USA).

### 4.2. Cell Culture

The cell lines THLE-3 (human normal liver cell line) and HepG2 (HB-8065) were obtained from the American Type Culture Collection (ATCC) and were cultured in DMEM, supplemented with 10% FBS and pen/strep (100 µg/mL) at 37 °C and 5% CO_2_.

### 4.3. Cell Viability Assay

The cell lines (1 × 10^4^ cells/mL) were exposed to 10-HDA at concentrations of 25, 50, 100, or 200 μg/mL and DOX (1 μM) in a 12-well plate at 37 °C and 5% CO_2_ for two days. Following addition of the MTT solution (50 μL) to the tested wells, the plate was incubated at 37 °C for 4 hours. The addition of 50 μL of dimethyl sulfoxide (DMSO) was used to stop the reaction, and the absorbance of the wells was read at 570 nm using a microplate reader (Bio-Rad Laboratories, Hercules, CA, USA) [18]. In all experiments, cells incubated with solvent (without any extract) were used as the control. Then, we determined the CC_50_ values for 10-HDA (the concentrations of 10-HDA that resulted in 50% cell viability).

### 4.4. The Morphological Study

To study the effect of 10-HDA on HepG2 cell morphology, the cells were treated with 10-HDA at the CC50 dose in 5% CO_2_ at 37 °C for two days. After washing with phosphate-buffered saline (PBS), they were assessed through an inverted microscope (Inverted Microscope, Optika, Italy). 

### 4.5. Annexin-V Assay

The initial and late apoptosis following the exposure of HepG2 cells to 10-HDA was determined by the AV assay using a commercial kit (APOAlert^®^ Annexin V; Clontech, Mountain View, CA, USA). Briefly, the HepG2 cells (1 × 10^5^ cells/mL) were treated with 10-HDA (at ½ CC50 and CC50) for 24 h. Following washing, cells were then labeled with AV-FITC and PI in. Finally, the assessment of apoptotic and necrotic cells was performed by a flow cytometer (BD Biosciences, San Jose, CA, USA).

### 4.6. Evaluating the Genes Expression by Real-Time PCR

Real-time PCR was used to determine the level of gene expression of miRNA-34a and some apoptosis genes, e.g., caspase-3, Bcl-2-associated X protein (BAX), and B-cell lymphoma protein 2 (Bcl-2). After extracting the ribonucleic acid (RNA) using Trizol reagent (Invitrogen, USA), a commercial kit from Qiagen, (Germantown, MD, USA) and the TaqMan MicroRNA reverse transcription kit were used for cDNA synthesis of apoptosis genes and miR-34a gene, respectively. Finally, SYBR green master mix was used to determine the expression level of the tested genes. The conditions of the thermal reaction were 94 °C for 10 min, and then 42 cycles of 94 °C for 12 s and 59 °C for 30 s. The 2-^ΔCt^ method was used to calculate the level of mRNA expression [17]. The sequences of the primer pairs used were: Bax F: 5′-GGCTGGACACTGGACTTCCT-3′, Bax R: 5′-GGTGAGGACTCCAGCCACAA-3′; Bcl-2 F: 5′-CATGCCAAGAGGGAAACACCAGAA-3′, Bcl-2 R: 5′-GTGCTTTGCATTCTTGGA TGAGGG-3′; Caspase-3 F: 5′-TTCATTATTCAGGCCTGCCGAGG-3′, Caspase-3 R: 5′-TTCTGACAGGCCATGTCATCCTCA-3′; and β-actin F: 5′-GTGACGTTGACATCCGTAAAGA-3′, β-actin R: 5′-GCCGGACTCATCGTACTCC-3′.

### 4.7. Western Blot

The cells (1 × 10^4^) were treated with various concentrations of 10-HDA for 24 h. Following lysing of the cells, the protein was extracted and measured by Bradford assay based on a protocol from a previous study [17]. The obtained proteins were resolved by 10% sodium dodecyl sulphate-polyacrylamide gel electrophoresis (SDS-PAGE) and transferred to a P(VDF-TrFE) piezoelectric membrane. Then, the membranes were blocked using 5% skimmed milk in 0.1% Tris-buffered saline with 0.1% Tween^®^ 20 detergent (TBST) at room temperature and then treated with monoclonal primary antibodies for poly [ADP-ribose] polymerase (PARP) (ab32064, Abcam, Waltham, MA, USA), Caspase-3 (ab32351, Abcam, USA), Caspase-9 (ab32539, Abcam, USA), Bcl2 (ab59348, Abcam, USA), or Bax (ab32503, Abcam, USA) with glyceraldehyde 3-phosphate dehydrogenase (GADPH) (ab8245, Abcam, USA) as the control. Next, the membranes were incubated with horseradish peroxidase (HRP)-conjugated secondary antibodies for 120 min at room temperature. The blots were visualized by means of chemiluminescence detection via the Amersham detection kit. 

### 4.8. Statistical Analysis

All experiments were done in triplicate. SPSS software version 22.0 (SPSS Inc., Chicago, IL, USA) was used for the analyses. *p* < 0.05 was set as statistically significant.

## 5. Conclusions

Although many chemical drugs, e.g., cisplatin, methotrexate, taxis, and doxorubicin, are used to treat HCC, there have been numerous reports related to the side effects of these drugs (e.g., emerging drug resistance, bone marrow failure, and gastrointestinal disorders). These issues have led scientists to search for novel anti-cancer drugs, mainly in natural products, that have greater efficiency and less toxicity. In recent years, growing attention has given to the benefits of food diets for treating and preventing various diseases and conditions such as cancers. Bee products (honey, RJ, and propolis) are famous valuable foods that have many health promoting and therapeutic benefits. Queen bee acid or 10-HDA is well-known as a lipid constituent or fatty acid in RJ and exhibits numerous pharmacological and therapeutic effects, e.g., antioxidant, anti-inflammatory, anti-tumor, immune boosting, and antimicrobial activities. Our results confirmed the potent in vitro cytotoxic effects of 10-HDA on HepG2 cells with no significant cytotoxic effects on normal cells. We found that 10-HDA markedly elevated the number of necrotic and apoptotic HepG2 cells. The real-time PCR results showed that the expression levels of the caspase-3, Bax, and miR-34a genes were significantly elevated. Contrary to these results, we found a significant reduction in the expression level of the Bcl2 gene. We also revealed that the levels of protein expression of Caspase-3, PARP, and Bax were markedly elevated following exposure of HepG2 cells to 10-HAD, while the level of protein expression of Bcl-2 was markedly reduced following exposure of HepG2 cells to 10-HAD. Therefore, although its mechanisms of action have not been fully studied, the induction of apoptosis via different pathways was determined as one of the principle mechanisms of action of 10-HDA against HepG2 cells. However, additional surveys must be performed to clearly investigate the mechanisms of action and safety of this fatty acid. 

## Data Availability

All data generated or analyzed during this study are included in this published article.
